# *COCOMAPS* 2.0: a web server for identifying, analyzing, and visualizing atomic interactions at the interface of biomolecular complexes

**DOI:** 10.1093/bioinformatics/btaf606

**Published:** 2025-12-03

**Authors:** Mohit Chawla, Utkarsh Kalra, Andrea Petta, Suraj Sharma, Abdul Rajjak Shaikh, Luigi Cavallo, Romina Oliva

**Affiliations:** Physical Sciences and Engineering Division, King Abdullah University of Science and Technology (KAUST), Thuwal 23955-6900, Saudi Arabia; Physical Sciences and Engineering Division, King Abdullah University of Science and Technology (KAUST), Thuwal 23955-6900, Saudi Arabia; Tagetik Software S.R.L, Lucca 55100, Italy; Department of Research and Innovation, STEMskills Research and Education Lab Private Limited, Faridabad 121002, Haryana, India; Department of Sciences and Technologies, University “Parthenope” of Naples, Naples 80143, Italy; Physical Sciences and Engineering Division, King Abdullah University of Science and Technology (KAUST), Thuwal 23955-6900, Saudi Arabia; Department of Sciences and Technologies, University “Parthenope” of Naples, Naples 80143, Italy

## Abstract

**Summary:**

Herein, we present COCOMAPS 2.0, for the analysis, visualization, and comparison of the interface in protein–protein and protein–nucleic acid complexes. COCOMAPS 2.0 complements the residue-level and buried surface area analyses of the original COCOMAPS tool with a comprehensive and accurate atomic-level characterization of the interface, enabling detailed interpretation of molecular recognition. Furthermore, it provides a greatly enhanced flexibility, interactivity, and efficiency in graphical visualizations.

**Availability and implementation:**

COCOMAPS 2.0 is accessible as a public web tool at https://aocdweb.com/BioTools/cocomaps2 and as a standalone code at https://doi.org/10.5281/zenodo.17390665.

At the core of many of the most important molecular processes in the cell, including signal transduction, electron transfer, gene expression and immune response, are interactions between biomolecules. Increasing evidence is revealing that perturbation of such interactions frequently leads to defective phenotypes. Mutations associated to human genetic disorders tend to alter the interaction between proteins more commonly than their folding and stability (Sahni *et al.* 2015, Cheng *et al.* 2021, Xiong *et al.* 2022). Further, disease-related mutations in DNA-binding proteins have been shown to cluster predominantly at the DNA interface, where they affect the interaction (Livesey and Marsh 2022, Osterburg *et al.* 2023).

An efficient visualization and analysis of the interface in the 3D structure of a biomolecular complex is therefore crucial for a full understanding of the functional and dysfunctional biological processes driven by the associated interactions, as well as for formulating testable predictions for interface modification and targeting (do Nascimento *et al.* 2024, Camps-Fajol *et al.* 2025).

In 2011, we presented COCOMAPS (bioCOmplexes COntact MAPS), a web tool using inter-residue contacts for the analysis of protein/nucleic acid complexes, and the first one to propose inter-residue contact maps for an efficient visualization of the interface (Vangone *et al.* 2011). Also due to its ease of use, COCOMAPS soon became popular and has remained so. It has been used for instance for complementing the analysis of the interface in newly solved experimental structures of large complexes (Li *et al.* 2017, Whitehead *et al.* 2023, Zinzula *et al.* 2024), as well as for a large-scale comparison of SARS-CoV-2 spike therapeutic antibody candidates (Schendel *et al.* 2025), to cite recent applications.

In the last decade, availability of structures of assemblies solved experimentally (Berman *et al.* 2000) or predictable at accuracy competitive with experimental structures has enormously increased (Lensink *et al.* 2023, Abramson *et al.* 2024). This motivated us to provide the community with a version 2.0 of COCOMAPS. In it, we put to use the understanding of the interface features we have been sharpening in our multiple participations as scorers in the CAPRI (Critical Assessment of PRedicted Interactions) experiment (Vangone *et al.* 2013, Lensink *et al.* 2016, Lensink *et al.* 2017, Lensink *et al.* 2018, Lensink *et al.* 2019, Barradas-Bautista *et al.* 2020, Lensink *et al.* 2023), and combine it with our expertise in detecting and energetically characterizing non-covalent interactions in biomolecules (Chawla *et al.* 2014, Chawla *et al.* 2015, Chawla *et al.* 2017, Kalra *et al.* 2020, Chawla *et al.* 2022).

Major advancements of COCOMAPS 2.0, as compared to COCOMAPS, include: (i) accepting input structures in the mmCIF file format; (ii) detecting atomic interactions and accurately classifying them in 16 distinct classes; (iii) providing an interactive Mol* visualization (Sehnal *et al.* 2021), which encompasses all the atomic interactions at the interface, and features hyperlinks for seamless navigation between 3D view and tables; (iv) supplying multiple representations of the interface, as a pie chart, a heatmap, a 2D and a 3D atomic contact map.

The core of the COCOMAPS 2.0 code lies in detecting atomic interactions at the interface, offering what is, to the best of our knowledge, the most comprehensive classification currently available among tools performing similar analyses (Jubb *et al.* 2017, Kayikci *et al.* 2018, Badaczewska-Dawid *et al.* 2022, Szulc *et al.* 2022, Del Conte *et al.* 2024, Schake *et al.* 2025). A comparative summary of the main features supported by such tools is provided in Table S1. The 16 types of interactions we classify are: H-bonds, salt bridges, weak H-bonds (CH-ON bonds), water-mediated contacts, metal-mediated contacts, disulfide bonds, halogen bonds, π–π/lone pair-π/anion-π/cation-π/amino-π/ONSH-π/CH-π interactions, polar and apolar vdW contacts. Most of these interactions are variably present across analogous tools, whereas metal-mediated, lone pair-π and amino-π contacts are not currently defined in any of them (see Table 1, available as supplementary data at *Bioinformatics* online). Additionally, contacts with interatomic distances below the sum of the respective vdW radii are classified as ‘clashes’; in case they meet the criteria for one of the interaction types listed above, this interaction type is reported and supplemented with an asterisk (*). Finally, contacts that fall within the selected cutoff inter-residue distance (5 Å by default), but do not correspond to any of the defined atomic contact classes, are referred to as ‘proximal’ (Jubb *et al.* 2017).

Classification of the interactions is based on criteria we carefully derived from the literature, including our own studies (Chawla *et al.* 2014, Chawla *et al.* 2017, Kalra *et al.* 2020, Chawla *et al.* 2022). Programs under the COCOMAPS 2.0 web tool have been written in Python 3.0, taking advantage of the numpy (v1.26.4), pandas (v2.2.2), scipy (v1.14.0), biopython (v1.83), and cctbx-base (v2024.5) libraries. Reduce (Word *et al.* 1999) is used for adding hydrogen atoms and HBPLUS (McDonald and Thornton 1994) for detecting hydrogen bonds, salt bridges, and water-mediated contacts. Other interactions are identified using in-house Python scripts. The selected parameters and thresholds were validated in previous studies and are reported in detail, with the corresponding references, on the About page of the web server (https://aocdweb.com/BioTools/cocomaps2/about). Accessible surfaces are calculated by NACCESS (Hubbard and Thornton 1993).

Inputs for COCOMAPS 2.0 are 3D structures, experimental or predicted, of complexes between protein, DNA and/or RNA molecular chains. Input files, in the PDB or the mmCIF format, can be directly retrieved from the wwPDB (with no size limit; extended PDB IDs are also supported) or uploaded locally (size up to 40 MB). Once a structure has been uploaded, the input page of COCOMAPS 2.0 will list the chain IDs for the molecules present in it and their respective range of residues and will allow the selection of the molecules (or part of them) involved in the interaction to be analyzed. In the advanced options, users may optionally also specify: a project name, a name for the molecules involved in the interaction and their email address, where to receive the output once the job is done. Users can also modify the cutoff distance for defining the residue-residue contacts and change, within a given range, thresholds for all the geometrical parameters (distances and angles) used to detect and classify the atomic interactions. After clicking the «Submit» button, users are redirected to the output page, which checks the job status and reloads until completion. Details on the runtime performance and scalability are also given on the web server About page.

COCOMAPS 2.0 outputs are displayed on the results web page for 3 months and archived as downloadable compressed files. A link to the online resource is also emailed to the user, if requested. At the top of the COCOMAPS 2.0 output, a Table of Atomic Interactions and a Mol* 3D view of the complex, with interacting residues in a ball-and-stick representation, are shown side by side (see Fig. 1). Each row of the Table reports a pair of interacting residues and lists all the atomic interactions between them. The table is sortable by residue number and filterable by interaction name/type. A full interactivity for seamless navigation between the Table and the 3D view is provided. When clicking a Table row, the corresponding residues will be zoomed in and highlighted in the Mol* visualization. By clicking the «+» button at the start of a row, all the atomic interactions between that pair of residues will be listed and detailed with names of the interacting atoms and relative distance/angle. By clicking the eye-shaped button next to an interaction type, the corresponding interactions will be zoomed and visualized as dashed lines in the Mol* visualization; the tag-shaped button activates labels on the interacting residues. On the other hand, by clicking on a residue in Mol*, the Table will be sorted to display at the top and highlight all the interactions involving that residue.

**Figure 1.**
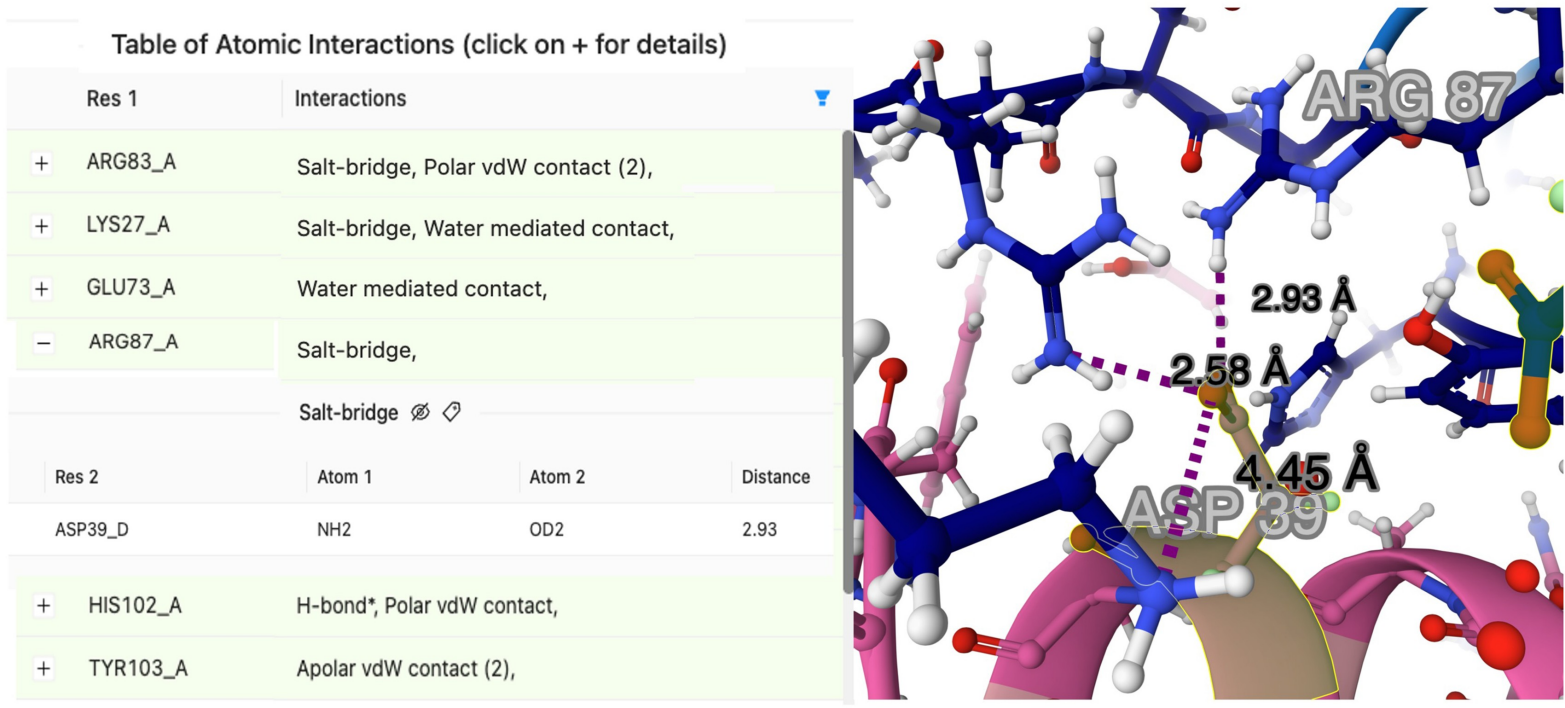
Details of the COCOMAPS 2.0 output for the interactions involving the barstar residue Asp39 in the barnase–barstar complex (PDB ID: 1x1u). *Left*: Table of Atomic Interactions; for the sake of readability the column reporting the Asp39 residue is not displayed here. *Right*: Mol* view; the three salt bridge interactions have been displayed and the interaction with Arg87 labeled. Table and Mol* star visualization are shown side by side, as they appear in the server output page.

As an example, in Fig. 1, the COCOMAPS 2.0 3D view and Table of Atomic Interactions are shown for the barnase–barstar complex (PDB ID: 1x1u), after clicking on the barstar residue Asp39 (chain D) at the interface. The table appears sorted to top list, highlighted in pale green, all the contacts involving Asp39. These are 11 specific atomic interactions with six different barnase residues, including 4 H-bonds, of which 3 are salt bridges, and 2 water-mediated contacts. For clarity, only the 3 salt bridge interactions have been shown in the 3D view and only that with Arg87 has been labeled. Both from the table and for the Mol* view, it is immediately clear that Asp39 is a hotspot for recognition. It is in fact the residue contributing most to the complex stability, with its single mutation to alanine causing a drop in the free energy of binding (ΔG) by 7.7 kcal/mol (Schreiber and Fersht 1995). For the sake of comparison, the COCOMAPS 1.0 output also reported the six Asp139 inter-residue contacts with barnase; however, among the 11 atomic interactions, only the 4 hydrogen bonds were listed, with no specification of salt bridges (see Fig. 1, available as supplementary data at *Bioinformatics* online).

The Mol* 3D visualization presents, in the top right corner, a selected Menu designed to resemble that in the «Explore in 3D» view of the wwPDB (Berman *et al.* 2000), to simplify the visualization experience to users already familiar with the wwPDB. It includes a «Screenshot/State snapshot» button, for taking high-definition pictures of the 3D view currently on the screen, a «Toggle expanding viewpoint button», for full-screen visualization of the complex, and Toggles for «Control panels», «Settings/Control info» and «Selection mode», from which experienced users can access all the advanced Mol* functionalities, such as sequence view and molecular component selection, modified molecular representation, high-quality rendering using lighting and outlines, plus additional geometric measurements (distances, angles, dihedrals) and labeling. Above the Mol* view, a «Download» button allows retrieval of the analysis results, while a «Reset» button enables users to return to the initial settings at any moment with a single click.

By scrolling down the output page, two tables are displayed, summarizing the interface. One table reports the number of residue-level interactions, while the other lists atomic interactions categorized by type. A hyperlink to the Mol* 3D view allows users to visualize all interactions of a selected type directly within the molecular representation of the complex.

Above these Summary tables, four clickable symbols connect to as many plots, providing a graphical overview of the interface. These are a pie chart, a heatmap, a 2D and a 3D contact map of the atomic interactions (see Fig. 2). For all of them, ready-to-print, high-resolution images, in the PNG, JPEG, PDF and SVG formats are provided. By hovering over the heatmap, details of the corresponding interactions are visualized. In the contact maps, all the specific types of interactions are displayed with different symbols and can be filtered in/out; the third dimension of the 3D map is in fact represented by the interaction type. All these graphical outputs are also interactive with the tabular data and the Mol* 3D view. At the bottom of the Summary tables/plots, an ASA Table reports information about the buried surface area upon complex formation, also detailed per residue, for each involved molecule.

**Figure 2.**
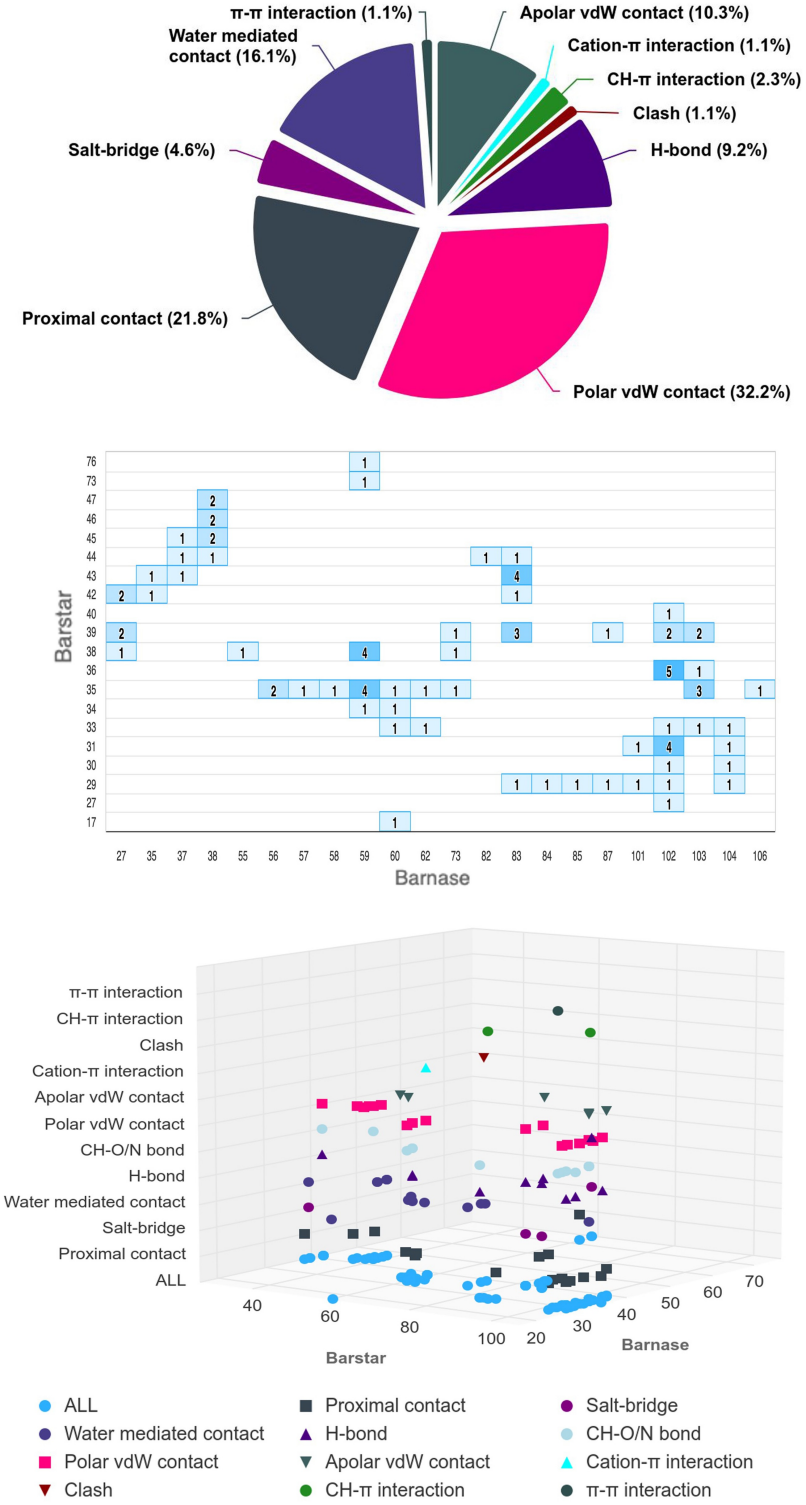
COCOMAPS 2.0 graphical overviews of the interface for the barnase–barstar complex (PDB ID: 1x1u). *Top*: Pie chart of the atomic interactions. *Middle*: Heatmap reporting the number of atomic interactions for each pair of interacting residues. *Bottom*: 3D atomic contact map: each type of interaction is displayed with a different symbol on a separate layer; hovering over the symbols, the identity of residues involved in the interaction is displayed.

In Fig. 2, the pie-chart, heatmap and 3D contact map summarizing the interface features are shown, again, for the barnase–barstar complex. The plots highlight the variety of atomic interactions at the interface and the large number of residues involved. It can also be noted that the interface is rich in strong electrostatic interactions. In fact, salt bridges, H-bonds and water-mediated contacts represent altogether almost one third of the interactions, accounting for the tight binding which makes this complex a model system for high-affinity protein–protein interactions (Lee and Tidor 2001).

To further illustrate the features and scope of COCOMAPS 2.0, we also applied it to the complex between Escherichia *coli* tRNA^Cys^ and cysteinyl-tRNA synthetase (CysRS). On the input page, for the tRNA chain, we selected only the anticodon residues (34–36) and, for the protein chain, the C-terminal anticodon-binding domain residues (403–461). The anticodon is in fact expected to play a major role in recognition, as substitutions within it, particularly at G34, are known to dramatically affect the CysRS cysteinylation (Komatsoulis and Abelson 1993). The Summary of interactions table and a Mol* 3D view from the COCOMAPS 2.0 output are shown in Fig. 3. Because of the residue range selection made, interactions in the table are specific to the anticodon. They include 15 directional atomic interactions (7 H-bonds, 3 water-mediated contacts, 3 CH–O/N bonds, 1 π–π and 1 CH–π contact), 8 of which involve G34. G34 interacts with four CysRS residues, through 2 H-bonds, 3 water-mediated contacts, 1 CH–O/N bond, 1 π–π and 1 CH–π contact (see Table 2, available as supplementary data at *Bioinformatics* online for details). The water-mediated contacts, shown in the 3D view of Fig. 3, are especially relevant as they create a hydration network involving both Asp436 and Arg423. Previously, only the G34 H-bonds with CysRS Arg427 and Asp436 and the π–π contact with Trp432 had been described (Hauenstein *et al.* 2004). To the best of our knowledge, no other public web server can both detect and interactively display in 3D all the atomic interactions occurring between G34 and CysRS (Table 2, available as supplementary data at *Bioinformatics* online). This example thus helps illustrate how COCOMAPS 2.0 expands the scope of similar tools by providing a more complete picture of molecular recognition, based on both strong and weak interactions, and on the fundamental hydration network, while requiring minimal effort from the user to extract and visualize desired information.

**Figure 3.**
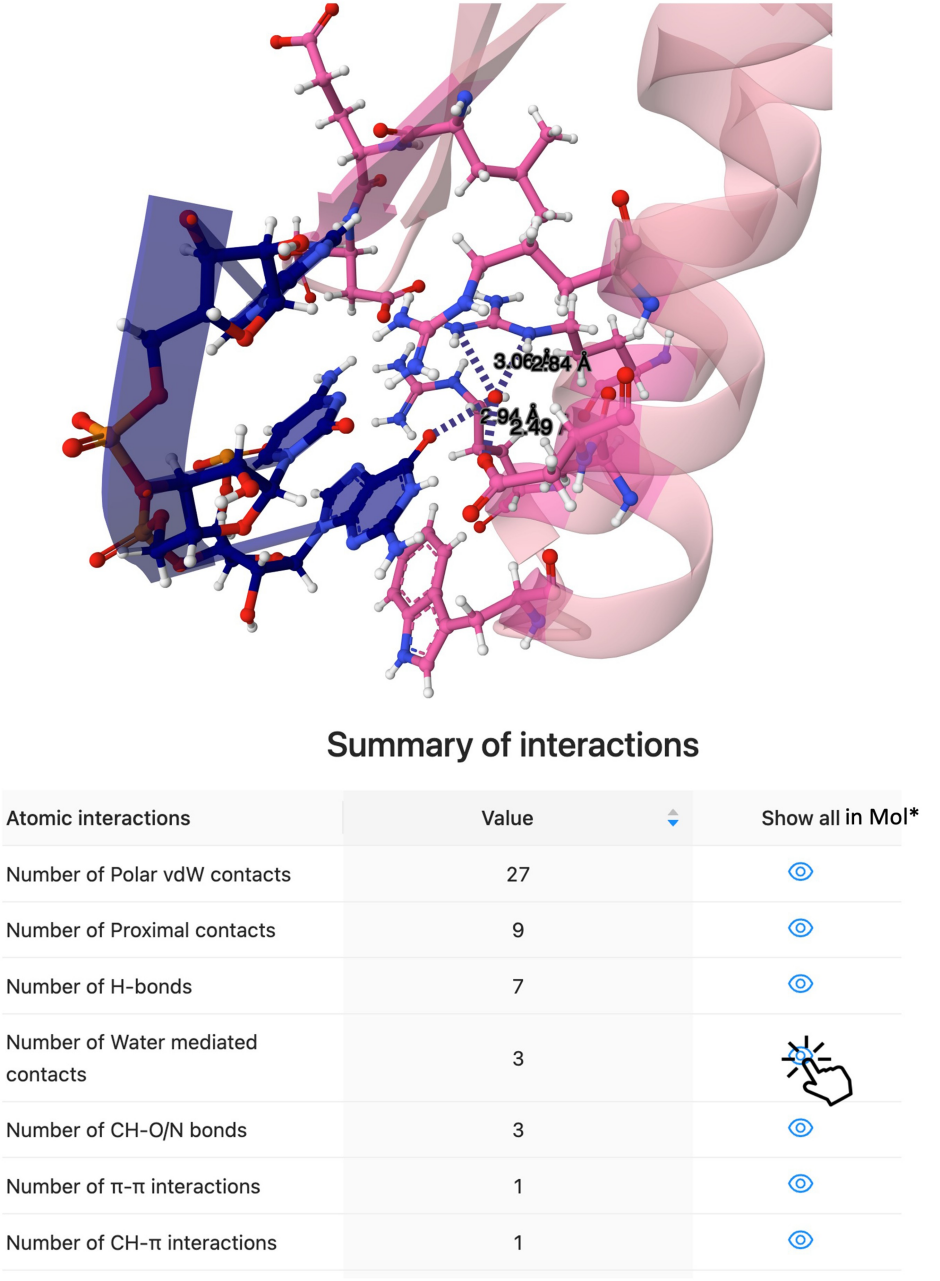
COCOMAPS 2.0 output for the interaction between the tRNACys GCA anticodon and the CysRS C-ter domain (PDB ID: 1u0b). *Top*: Mol* view of the complex, where only the water-mediated contacts, all involving G34, are displayed. *Bottom*: Summary of interactions table. The water-mediated contacts can also be displayed in Mol* with a simple click on the eye-shaped button here.

In conclusion, as shown above, COCOMAPS 2.0 is designed to offer an intuitive, informative and productive experience, regardless of the user’s familiarity with biomolecular structures or the chemical nature of interactions. The accuracy and comprehensiveness of the atomic interactions classification, combined with full interactivity between tabular information, graphical outputs and 3D visualization, make COCOMAPS 2.0 useful for a wide range of users—from undergraduate students exploring molecular interfaces for the first time to experienced structural biologists conducting detailed and informed analyses of physico-chemical interactions.

## Supplementary Material

btaf606_Supplementary_Data

## Code availability

COCOMAPS 2.0 is accessible as a public web tool at https://aocdweb.com/BioTools/cocomaps2 and as a standalone code at https://doi.org/10.5281/zenodo.17390665.
