# Supplementary material for: COCOMAPS 2.0: a web server for identifying, analyzing, and visualizing atomic interactions at the interface of biomolecular complexes
Source: Bioinformatics. 2025 Dec 3;41(12):btaf606. doi: 10.1093/bioinformatics/btaf606 (PMC12684709; doi:10.1093/bioinformatics/btaf606)
Supplement: btaf606_Supplementary_Data [file btaf606_supplementary_data.pdf]

Supplementary Materials to:

COCOMAPS 2.0: a web server for identifying, analyzing and visualizing atomic interactions at the interface of biomolecular complexes

Mohit Chawla,[1,*] Utkarsh Kalra,[1] Andrea Petta,[2] Suraj Sharma,[3] Abdul Rajjak Shaikh,[4] Luigi Cavallo,[1] and Romina Oliva [4,*]

[1] Physical Sciences and Engineering Division, King Abdullah University of Science and Technology (KAUST), Thuwal 23955-6900, Saudi Arabia.
[2] Tagetik Software S.R.L, Via Franklin Delano Roosevelt, 55100 Lucca, Italy.
[3] Department of Research and Innovation, STEMskills Research and Education Lab Private Limited, Faridabad, Haryana 121002, India.
[4] Department of Sciences and Technologies, University “Parthenope” of Naples, Centro Direzionale Isola C4 80143, Naples, Italy.

-Table of minimum distances

Molecule1				Molecule2				Distance	aaProperty	
Res1	N° res1	Atom1	Chain1	Res2	N° Res2	Atom2	Chain2	Dist (A)	Property1	Property2
Filter: All	Filter: All	Filter: All	Filter: All	Filter: All	Filter: All	Filter: All	Filter: All		Filter: All	Filter: All
LYS	27	NZ	A	ASP	39	OD1	D	4.45	phil	phil
GLU	73	OE1	A	ASP	39	OD1	D	4.54	phil	phil
ARG	83	NH2	A	ASP	39	OD1	D	2.58	phil	phil
ARG	87	NH2	A	ASP	39	OD2	D	2.93	phil	phil
HIS	102	NE2	A	ASP	39	OD2	D	2.90	phil	phil
TYR	103	OH	A	ASP	39	CG	D	3.31	phil	phil

-H-Bonds Table

ACCEPTOR				DONOR				Dist(A)	TYPE	
Chain1	Res1	N° res1	Atom1	Chain2	Res2	N° Res2	Atom2	Dist (A)	Atoms category	Dist CA-CA
Filter: All	Filter: All	Filter: All	Filter: All	Filter: All	Filter: All	ASP	Filter: All		Filter: All	Filter: All
A	83	ARG	NH1	D	39	ASP	OD1	2.95	SS	10.00
A	83	ARG	NH2	D	39	ASP	OD1	2.58	SS	10.00
A	87	ARG	NH2	D	39	ASP	OD2	2.93	SS	10.58
A	102	HIS	NE2	D	39	ASP	OD2	2.90	SS	8.72

Figure S1. Details of the COCOMAPS 1.0 output for the interactions involving the barstar residue Asp39 in the barnase-barstar complex (PDB ID: 1x1u).

Table S1. Comparison between the main features of COCOMAPS 2.0 and those of similar tools. Features common to all tools are highlighted in gray, while features unique to COCOMAPS 2.0 are highlighted in pale green. “Protein” is abbreviated as “Pr” and “Nucleic acid” as “NA” in the table.

	COCOMAPS 2.0	Arpeggio	Protein Contacts Atlas	fingeRNAt	PLIP 2025	RING 4.0	MAPIYA
**Macromolecular Interaction**	Pr-Pr<br>Pr-NA<br>NA-NA	Pr-Pr<br>Pr-NA<br>NA-NA	Pr-Pr<br>Pr-NA<br>NA-NA	Pr-NA<br>NA-NA	Pr-Pr<br>Pr-NA	Pr-Pr<br>Pr-NA<br>NA-NA	Pr-Pr<br>Pr-NA<br>NA-NA
**File Format**	PDB<br>mmCIF<br>extended PDB	PDB	PDB	PDB<br>SDF (ligands)	PDB	PDB<br>mmCIF<br>single & multi-state	PDB
**Interface Selection**	Yes	No[&]	No	Yes	No	No	Yes
**Residues Range Selection**	Yes	Yes[&]	No	No	No	No	No
**Interactive 3D Visualization**	Yes<br>(Mol*)	Yes<br>(WebGL)	Yes	No<br>(Pymol plugin)	Yes<br>(JSmol)	Yes<br>(Mol*)	Yes<br>(Mol*)
**2D Contact Maps**	Yes	No	No[%]	No	No	Yes	Yes
**3D Contact Maps**	Yes	No	No	No	No	No	No

	COCOMAPS 2.0	Arpeggio	Protein Contacts Atlas	fingeRNAt	PLIP 2025	RING 4.0	MAPIYA
**Other Charts/Visualizations**	Pie chart<br>Heatmap	-	Secondary structure<br>Chord plot<br>Matrix plot<br>Asteroid plot	-	-	Interaction graph	interaction network diagram
**Detection of Atomic Interactions**	Yes	Yes	Yes	Yes	Yes	Yes	Yes
**# of Interactions Detected**	16+2	15+1	14§	9+6	8	10	10
**Notes on Detected Interactions**	+2 refers to proximal contacts and clashes	+1 refers to proximal contacts; of the 15 Interactions above, "polar" and "weak polar" contacts correspond to H-bonds and weak H-bonds without angle terms, being less sensitive to H placement	Details about interactions given, but only classification at the molecular level provided, i.e. L-S (Ligand-Substrate) or L-W (Ligand-Water).	+6 refers to the possibility of adding interactions using a plugin<br>Interactions reported as present/absent, no details given	Missing info about residue name and number of the second molecule	Details about interactions given, to be parsed by users from the overall tables divided by contact type	Interactions with details visualized in the 2D contact map, not reported in tables
**Hydrogen bonds**	Yes	Yes	Yes	Yes	Yes	Yes	Yes
**CH—O/N bonds**	Yes	Yes	Yes	No	No	No	No

	COCOMAPS 2.0	Arpeggio	Protein Contacts Atlas	fingeRNAt	PLIP 2025	RING 4.0	MAPIYA
**Halogen bonds**	Yes	Yes	Yes	Yes	Yes	Yes	No
**Salt bridges**	Yes	Yes	Yes	Yes	Yes	Yes	Yes
**Metal-mediated contacts**	Yes	No (only metal coordination)	No (only metal coordination)	No (only metal coordination)	No	No	No
**Water-mediated contacts**	Yes	Yes	No	Yes	Yes	No	No
**S-S bonds**	Yes	No	No	No	No	Yes	No
**π-π interactions**	Yes	Yes	Yes	Yes	Yes	Yes	Yes
**Cation-π interactions**	Yes	Yes	Yes	Yes	Yes	Yes	Yes
**Anion-π interactions**	Yes	No	No	Yes	No	No	Yes
**Lone pair-π interactions**	Yes	No	No	No	No	No	No
**Amino-π interactions**	Yes	No	No	No	No	No	No
**O/N/SH-π interactions**	Yes	Yes	Yes	No	No	Yes	Yes
**CH-π interactions**	Yes	Yes	Yes	No	No	No	No
**Polar vdW contacts**	Yes	Yes	Yes	No	Generic vdW	Generic vdW	No

	COCOMAPS 2.0	Arpeggio	Protein Contacts Atlas	fingeRNAt	PLIP 2025	RING 4.0	MAPIYA
**Apolar vdW contacts**	Yes	Yes	Yes	No	Generic vdW	Generic vdW	No
**User-defined interactions**	No	No	No	Yes	No	No	No
**Accessible Surface Area Statistics**	Yes	No	No	No	No	No	Yes
**BSA of the complex**	Yes	No	No	No	No	No	No
**BSA of individual residues in both chains**	Yes	No	No	No	No	No	No
**Software features**							
Custom thresholds	Yes	No	No	Yes	Yes	Yes	Yes
Last update (Year)	2025	2016	2018	2022	2025	2024	2022
**Availability**							
Command line	Yes	Yes	Yes	Yes	Yes	Yes	No
Web server	Yes	Yes	Yes	No	Yes	Yes	Yes

% A matrix plot, somewhat like a 2D map, is provided.

& Although two chains and/or subsets of residues can be selected, contacts provided and displayed report together intra- and inter-chain interactions.

§ Arpeggio used to calculate contacts.

Table S2. Comparison of the atomic interactions detected between $tRNA^{Cys}$ G34 and CysRS residues.

G34-interacting residue	COCOMAPS 2.0	Arpeggio	Protein Contacts Atlas	PLIP 2025	RING 4.0	MAPIYA
**Arg423**	Water mediated contact (2), Polar vdW contact	Water mediated contact (2)[a]	1 L-S	H-bond	-	H-bond, salt-bridge, ion-dipole, cation-π
**Arg427**	H-bond, CH-O/N bond, Polar vdW contact (5)	H-bond,[b] CH-O/N bond[b]	10 L-S	H-bond	H-bond, vdW	H-bond, salt-bridge, ion-dipole, cation-π
**Trp432**	π-π interaction, CH-π interaction	π-π interaction,[a,b] CH-π interaction,[b] Aromatic contacts (2)[b]	19 L-S	π-π interaction	π-π interaction	H-bond, anion-π, π-π stacking
**Ala435**	Proximal contact	-	-	-	-	-
**Asp436**	H-bond, Water mediated contact, Polar vdW contact (3)	H-bond(2),[b] Water mediated contact[a]	9 L-S	Salt-bridge	H-bond, vdW	H-bond, ionic repulsion, ion-dipole, anion-π, π-π stacking
**Arg439**	Proximal contact	-	-	-	-	H-bond, salt-bridge, ion-dipole, cation-π, π-π stacking
**Asp451**	Proximal contact	-	-	-	-	H-bond, ionic repulsion, ion-dipole, cation-π, π-π stacking
**Water529**	-	H-bond with water (3)[b]	3 L-W	-	-	-

**Notes on How Interactions are Reported and Visualized**	Information on atomic contacts available in a table sortable by residue number and interactive with the 3D view. All the details about interactions (type, atoms involved, distances, angles) are provided.<br>Each and all the interactions can be selectively displayed in the web server 3D view.	Information on atomic contacts was visually extracted from the PyMol .pse files provided in output from the overall complex analysis (a) and upon selecting the G34 residue only (b). Downloaded textual contact list (.contacts file) provided a binary output, not easily to convert to a class definition.<br>The atomic contacts are not displayed interactively on the web server but shown all together (with intra-chain contacts) in the PyMol .pse file.	Information on atomic contacts to be parsed from the overall interchain textual contact list, to be downloaded, which provides details about atoms involved in the interactions and relative interatomic distance but offers no classification, other than that at molecular level, i.e. L-S (Ligand-Substrate) or L-W (Ligand-Water). Contacts involving water molecules do not bridge atoms of the two chains and therefore cannot be considered water-mediated interactions.<br>Interactions are not interactively displayed in the 3D view of the complex.	Information on atomic contacts to be parsed from the overall interchain textual contact lists, divided by contact type, which is also shown on the web site output page.<br>Only the atom number (no atom name, residue name & number) is given for the nucleotides, making complex the extraction of information on interactions involving specific RNA residues, such as G34.<br>Interactions are not displayed in the online or downloadable 3D views of the complex.	Information on atomic contacts to be parsed from the overall interchain textual contact lists, divided by contact type, which is also shown on the web site output page.<br>Interactions of G34 with CysRS can be displayed all together, but not selectively, in the web server 3D view of the complex.	All contacts are reported as “possible” and obviously include false positives; this is because the assignment is not specific but mainly based on a distance cut-off. A cut-off of 5 Å was set here for the analysis.<br>Information on atomic contacts had to be extracted from the contact maps reported in output by hovering over them.<br>Interactions are not interactively displayed in the 3D view of the complex.
